# Infrared Photodissociation Spectroscopy of Cationic Nitric Oxide Clusters, [(NO)_*n*_]^+^, and [NO_2_(NO)_*n*_]^+^

**DOI:** 10.1021/acs.jpca.5c01377

**Published:** 2025-04-21

**Authors:** Peter D. Watson, Gabriele Meizyte, Philip A. J. Pearcy, Edward I. Brewer, Alice E. Green, Anthony J. Stace, Stuart R. Mackenzie

**Affiliations:** † Department of Chemistry, Chemistry Research Laboratory, University of Oxford, Mansfield Road, Oxford OX1 3TA, U.K.; ‡ School of Chemistry, University Park, University of Nottingham, Nottingham NG7 2RD, U.K.

## Abstract

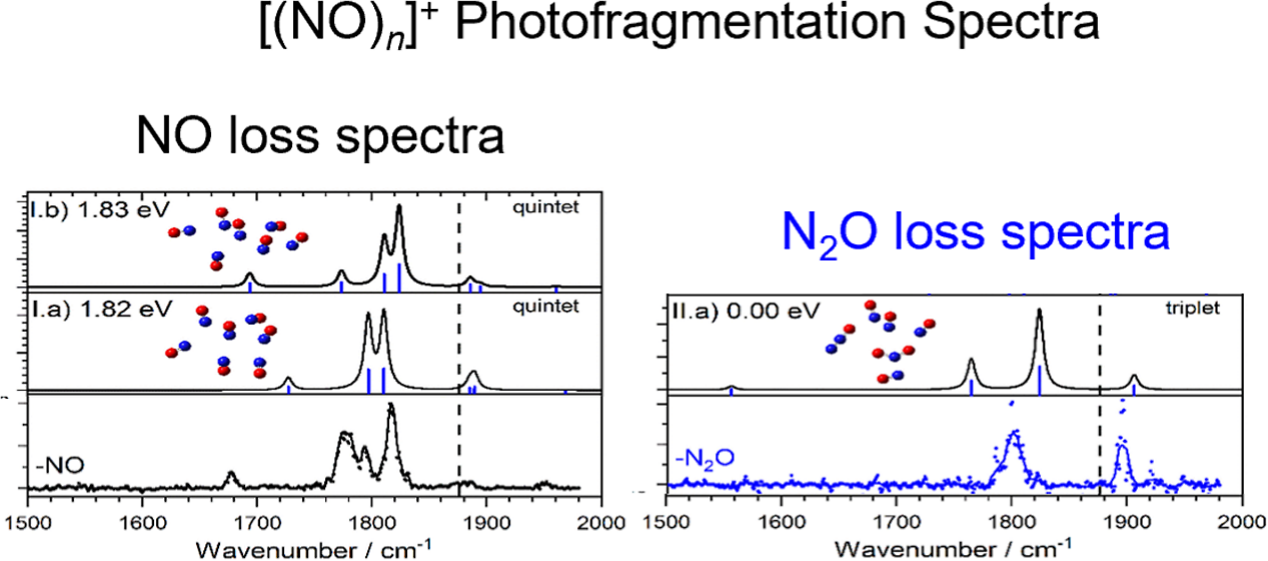

Photofragmentation spectroscopy provides a powerful method for the determination of structures and bonding in isolated gas-phase clusters. Here we report infrared action spectra of mass-selected cationic nitric oxide clusters, (NO)_*n*_^+^ (*n* = 3–8), and mixed NO_2_(NO)_*n*_^+^ clusters which are interpreted with the help of quantum chemical calculations. Despite the rich potential energy landscape which exhibits very many calculated low-energy isomers, clear structural motifs are observed. Important differences between our (NO)_*n*_^+^ spectra and others published previously are interpreted in terms of the qualitatively different experimental techniques employed in the initial formation of the clusters in each study. Finally, spectra recorded in different fragmentation channels provide clear evidence for intracluster chemistry leading to the formation of mixed nitrous oxide/nitrogen dioxide/nitric oxide complexes, (N_2_O)(NO_2_)(NO)_*n*_^+^.

## 1 Introduction

Nitric oxide, NO, is a deceptively simple molecule whose widespread chemical and biological importance earned it the accolade of Science magazine’s molecule of the year 1992.^1^ Not only do all three simple charge states (NO, NO^+^ and NO^–^) play essential roles in a variety of key physiological and neurological functions^2,3^ but nitric oxide is also a common atmospheric pollutant, harmful by inhalation and, alongside other NO_*x*_ gases, subject to emission controls.^4,5^ Both NO and NO^+^ are found in the upper atmosphere and play roles in ozone depletion and acid rain chemistry. Molecular clusters involving nitric oxide thus have implications for modeling of atmospheric kinetics. The cationic dimer, (NO)_2_^+^ is strongly bound (*D*_0_ = 0.59 ± 0.02 eV)^6^ suggesting reasonable lifetimes in lower pressure regions.

The open shell (X ^2^Π) nature of ground state NO leads to it clustering easily, both with itself and with other small molecules and, as a result, a rich literature of mass spectrometric, spectroscopic and theoretical studies exists. Our focus here is specifically on the spectroscopy of cationic NO clusters, [(NO)_*n*_]^+^, whose properties, including production efficiency, often exhibit pronounced odd–even alternations reflecting the fact that even (odd) *n* clusters have odd (even) numbers of electrons with odd *n* clusters the more stable.^7,8^ The smallest “cluster”, (NO)_2_^+^, has been studied in the gas-phase,^9−15^ in solid matrices,^13,16−18^ and computationally.^19^ In solid neon, the *cis*-ONNO^+^ vibrational band (at 1619 cm^–1^) has a markedly higher frequency than the corresponding *trans*-ONNO^+^ (1424 cm^–1^).^18^ Of particular relevance to the present work, one of us (Stace) has previously reported line-tunable CO laser IR dissociation of (NO)_*n*_^+^ and rare-gas tagged Ar-, Kr-, Xe-(NO)_*n*_^+^ clusters produced by electron impact ionization of the neutral cluster.^15,20^ In the case of Ar-(NO)_2_^+^, a partially resolved double band feature at 1694 and 1703 cm^–1^ was observed and tentatively interpreted as the cis and trans isomers.^15,20^ Tagging with rare-gas atoms was found to help thermalize otherwise internally hot (NO)_*n*_^+^ clusters but led to marked spectral shifts in the spectra of several species as well as alternative fragmentation pathways (NO vs rare gas loss). In common with findings from other studies,^8^ the (NO)_3_^+^ was found to represent a stable core structure to which subsequent moieties add. Stace and co-workers have also applied their IR photofragmentation studies to a range of other (NO)_*n*_^+^·X (X = SF_6_, C_2_H_4_, O_2_, CO_2_, CH_3_Cl, and CF_4_) species.^20−24^

The open shell nature of NO presents a number of challenges for quantum chemical calculations of its clusters. In many cases single reference density functional theory, though tractable in terms of computational cost, struggles in predicting the electronic properties of a multiple low-lying electronic states for all but the smallest cluster sizes.^19,25−28^ In many cases these shortcomings are met using configuration interaction (CI)^29^ and multireference methods^30−33^ which perform well in comparison to experiment.

We have recently reported the infrared action spectroscopy of gas-phase metal nitrosyl complexes, M^+^(NO)_*n*_ (M = Co, Rh, Ir),^34^ during the course of which we generated significant populations of both [(NO)_*n*_]^+^ and [NO_2_(NO)_*n*_]^+^ (*n* = 3–9) complexes. We report here the action spectra of these species in the region 1500–2100 cm^–1^ and compare them with quantum chemical simulations of low-lying structural isomers. Where possible, we compare our spectra with those reported previously for the same species and observe significant differences which we interpret as arising from the different cluster sources employed.

## 2 Experimental and Computational Method

**Figure 1:**
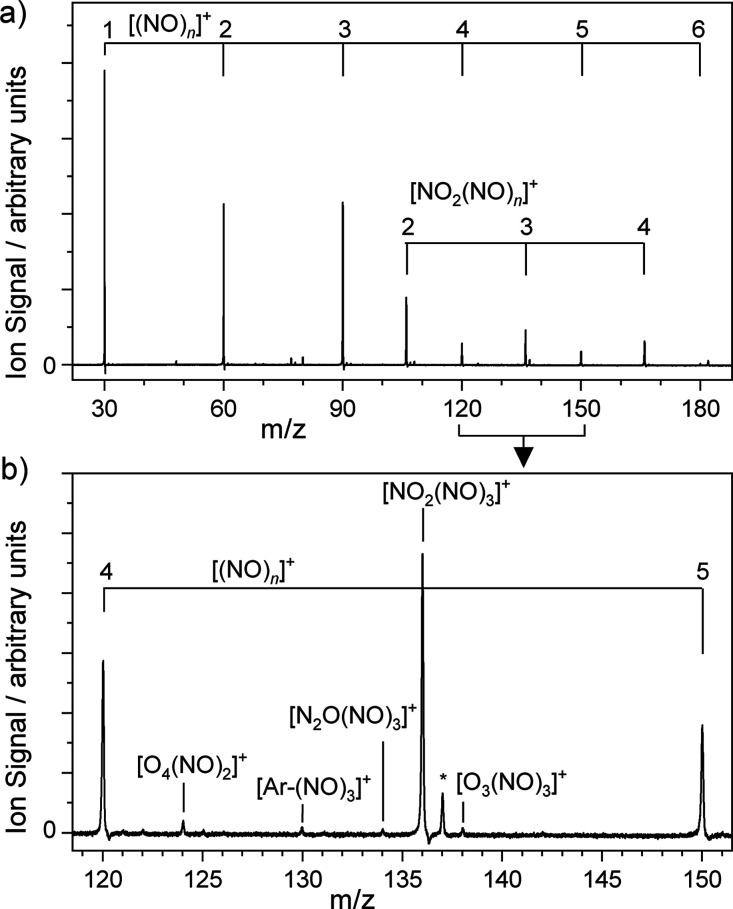
(a) Time-of-flight (ToF) mass spectrum of [(NO)_*n*_]^+^ and [NO_2_(NO)_*n*_]^+^ complexes produced by laser ablation of a metal target in the presence of a carrier gas comprising 1.5% NO in Ar. (b) Detailed view of the ToF spectrum between *m*/*z* = 120–150 illustrating a variety of molecular species. The asterisk, *, indicates cluster species involving residual H_2_O contamination.

The experimental setup used in these experiments has been described in detail previously.^34−37^ In this case, (NO)_*n*_^+^ clusters were generated alongside metal-nitrosyl, (M-NO)_*n*_^+^, clusters described in previous work.^34^ Briefly, a rotating metal target is ablated by a 532 nm pulse from an Nd:YAG laser (ca. 2–10 mJ, 10 Hz, 8 ns pulse), in the presence of a pulsed supersonic expansion of NO in Ar (NO v/v: 1.5%). The resultant molecular beam is skimmed and then passes through a quadrupole mass filter and quadrupole bender before being extracted into a reflectron time-of-flight (ReToF) mass spectrometer. Figure 1 shows a typical mass spectrum recorded with the quadrupole operating in rf-only mode (i.e., without mass selection). Strong [(NO)_*n*_]^+^ and [O(NO)_*n*_]^+^ (assigned on the basis of fragmentation patterns to [NO_2_(NO)_*n*−1_]^+^ ion signals were found with laser ablation markedly earlier in the gas pulse than typically employed for generating metal-containing complexes.^38,39^ We believe the initial NO^+^ is generated by electron impact from electrons emitted from the metal surface during ablation. In this sense it is similar to approaches commonly used for generating anionic complexes.^40^ Note that we employ the convention of [(NO)_*n*_]^+^ notation (i.e., square brackets) to indicate that species are assigned solely on the basis of their mass. Clearly (NO)_*n*_^+^ and (N_2_O)(NO_2_)(NO)_*n*−3_^+^ have identical mass and spectroscopy and/or fragmentation patterns are required to assign structures. Indeed, although the former dominates there is evidence for the presence of both structural forms (see Section 3.3) below).

Here, the initial NO ionization is assumed to by electron impact ionization in, or as a result of, the plasma generated in the metal ablation and provides the initial nucleation site for subsequent clustering within the molecular beam
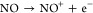
1
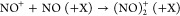
2
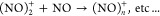
3in which X is some third body (almost certainly an Ar atom) required for efficient clustering. We cannot rule out the four-photon nonresonant multiphoton ionization of NO as the original NO^+^ source but, given the cross-section for this process at 532 nm is (7.1 ± 0.1) × 10^–117^ cm^8^ s^7^ this seems unlikely at these laser intensities (∼1 × 10^8^ W/cm^2^).^41^

The time-of-flight mass spectrum is dominated by [(NO)_*n*_]^+^ and [NO_2_(NO)_*n*_]^+^ clusters, the exact distribution of which depends on NO partial pressure, backing pressure and ablation laser timing. In our source, ablation occurs in the throat of the expansion where the number density is high enough for clustering collisions before cooling. Under all conditions, the relative ion intensities of the [(NO)_*n*_]^+^ clusters exhibit a slight odd–even alternation, with odd *n* clusters more intense than the corresponding even *n* clusters, reflecting the relative thermodynamic stability of the former.^8^ More weakly represented in the mass spectrum are Ar-(NO)_*n*_^+^ clusters and a number of oxygen-rich species assigned on the basis of their mass as [O_4_(NO)_2_]^+^, [N_2_O(NO)_3_]^+^, and [O_3_(NO)_3_]^+^. We interpret the presence of these species as resulting from intracluster reactions—presumably forming heterogeneous clusters like [(N_2_O)(NO_2_)(NO)_*n*_]^+^ and/or [(N_2_)(O_2_)(NO)_*n*_]^+^ with subsequent N_2_O or N_2_ loss. Unfortunately, the signals of many of these species are too weak to record infrared spectra. The quadrupole mass filter permits mass selection of individual species in the mass spectrum, albeit at the cost of transmission efficiency.

Infrared photodissociation (IR-PD) spectroscopy of mass-selected ions is performed using the counterpropagating output of a tunable infrared optical parametric oscillator/optical parametric amplifier (OPO/OPA, Laservision, scanning between 1400 and 2100 cm^–1^) before the reflectron extraction region. IR-PD spectra are generated by monitoring the relative parent and daughter fragment ion signals in the presence and absence of the infrared pulse and presented, unless otherwise stated, as relative normalized cross sections, σ, for the daughter channel using a modified Beer–Lambert law^42^
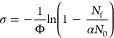
4where *N*_f_ and *N*_0_ are the daughter and parent ion signals respectively, with *N*_0_ measured in the absence of the IR laser. α is an instrumental response function representing the overlap between the cluster beam and the infrared pulse (taken here to be 1), and Φ is the photon flux (proportional to the IR pulse energy). In this work, the infrared laser operated at 5 Hz so that alternate cluster beam pulses were interrogated.

To assist in assigning the experimental spectra, DFT calculations of energetically low-lying structures were performed using the Gaussian 16 software package.^43^ Calculating NO cluster structures represents a significant challenge with respect to charge localization, large conformer spaces for all but the smallest clusters and likely multireference character.^44^ The calculations involved here are significantly more involved than those of our previous study of metal nitrosyl complexes (in which the charge is predominantly localized on the metal center),^34^ and required consideration of charge localization. These include a monomer-based cluster in which charge is local to a single NO monomer, charge local to a subset of the cluster (i.e., an [(NO)_2_]^+^ dimer), and charge being delocalized over the cluster.^45^

The CAM-B3LYP functional was used for geometry optimizations with Dunning augmented, correlation consistent basis sets of triple-ζ quality (denoted aug-cc-pVTZ) with harmonic frequency analysis confirming geometric minima.^46−48^ To sample the conformer space adequately a modified *Kick*^3^ algorithm was used to generate starting guesses from sequentially solvated [(NO)_*n*_]^+^ clusters.^49,50^ For small *n*, solvation of specific, known structural motifs (e.g., dimer and trimer) was also considered. For example, for *n* = 5, both the (NO)_4_^+^ + NO and (NO)_3_^+^ + (NO)_2_ structures were considered. For ease of comparison with experimental data, computed harmonic frequencies are scaled with respect to the “free” NO fundamental stretch (1876 cm^–1^, scaling factor = 0.9268)^51^ and spectral lines convoluted with a Lorentzian function (fwhm = 8 cm^–1^). For [NO_2_(NO)_*n*_]^+^ clusters, the asymmetric NO_2_ stretch is scaled independently against the corresponding “free” NO_2_ stretching frequency (1616 cm^–1^, scaling factor = 0.9252)^52^ as done in recent studies of mixed-ligand complexes.^37,53,54^ The choice of scaling factor in homogeneous clusters is not trivial but scaling instead to the NO^+^ fundamental (2344 cm^–1^, scaling factor = 0.9265) would make little difference.

## 3 Results and Discussion

### 3.1 Infrared spectra of [(NO)_*n*_]^+^ clusters

**Figure 2:**
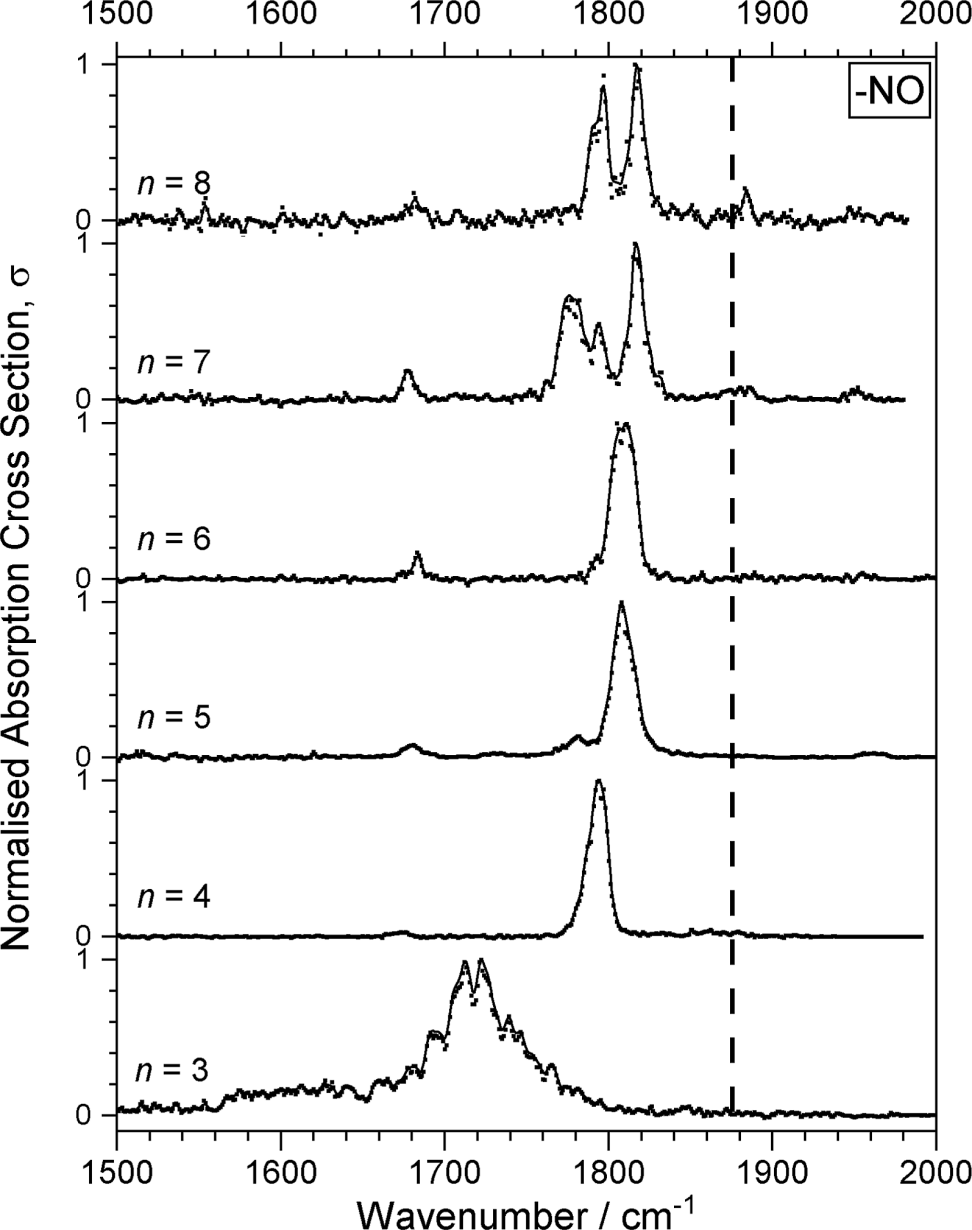
Infrared photodissociation (IR-PD) spectra of the [(NO)_*n*_]^+^ clusters for *n* = 3–8, recorded in the NO loss channel (similar spectra recorded in other fragmentation channels are shown in Figure S1). Cross sections have been normalized to the maximum intensity and a 10 cm^–1^ running average has been applied. The dashed line marks the fundamental band in free NO at 1876 cm^–1^.^55^

An overview of the IRPD spectra recorded for the [(NO)_*n*_]^+^ (*n* = 3–8) clusters is shown in Figure 2. In each case the spectrum shown was recorded in the simple NO loss channel (i.e., for the [(NO)_*n*_]^+^ + h*v* → [(NO)_*n*-1_]^+^ + NO process). Spectra recorded in other fragmentation channels are provided in the Supporting Information. These typically involve loss of multiple NO moieties (Figure S1) but also, weakly, N_2_O loss (see Section 3.3 below and Figure S3). Consistent with previous work,^15^ the trimer cation, [(NO)_3_]^+^, is the smallest cluster for which fragmentation was observed and its spectrum is distinct from that of all larger clusters comprising a broad, largely unresolved band centered near 1720 cm^–1^. Our spectrum is similar to that reported by Mouhandes and Stace for the same species, albeit red-shifted by ca. 60 cm^–1^.^15^ Our band lies much closer to the spectral position of the sharper band observed for Ar loss from Ar(NO)_3_^+^ (see direct comparison below).^15^ Broad vibrational bands in IR-PD spectra are often the signature of multiple rather than single-photon absorption which is consistent with the relative stability of the trimer cation for which Hiraoka et al. report the enthalpy change for NO loss as 12.8 kcal mol^–1^ (0.56 eV),^8^ three times that of (NO)_4_^+^ and more than twice the value for (NO)_5_^+^.

The spectra for larger complexes (*n* > 3) are dominated by a strong band around 1800 cm^–1^ red-shifted from the free NO stretch at 1876 cm^–1^,^55^ which splits into several bands for *n* = 7, 8, possibly due to multiple stable structural motifs including secondary solvation shells or as a result of intracluster reactions (see Section 3.3).

**Figure 3:**
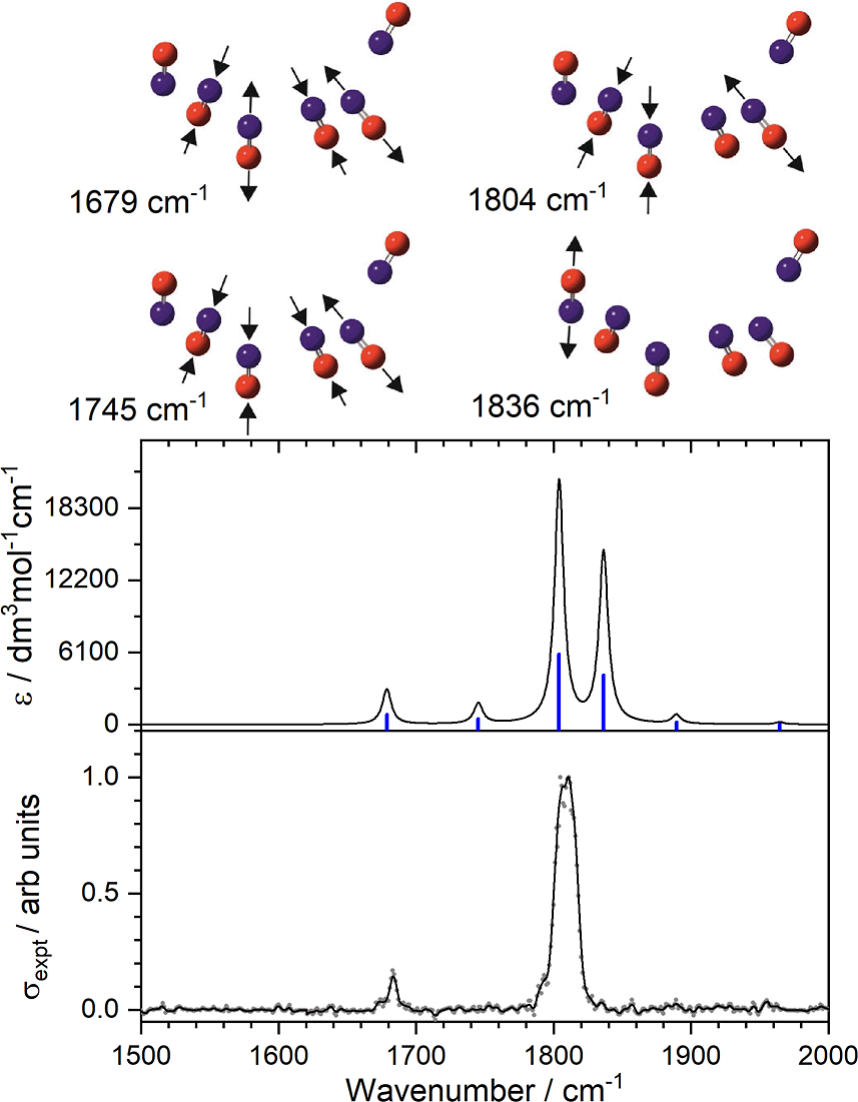
Comparison of experimental and representative simulated infrared action spectra for the [(NO)_6_]^+^ cluster. Vibrational modes observed comprise a series of coupled in-phase and out-of-phase NO stretches plus one highly localized NO stretch (1836 cm^–1^) as predicted by CAM-B3LYP/aug-cc-pVTZ calculations.

In addition to the main vibrational bands 1800 ± 50 cm^–1^ weaker satellite bands are clearly observed in the spectra of complexes *n* > 3, especially 1650–1700 cm^–1^ but also to the blue of the main peaks, at 1960 cm^–1^ for *n* = 5, red shifting with *n* to 1880 cm^–1^ by *n* = 8. These bands are reproduced in the simulations and arise from stretches in the cluster core. For smaller clusters with a trimer core the central NO molecule vibrates. For larger clusters (i.e., *n* ≥ 6), this geometric feature is best described as a chain of two NO dimers solvated by additional NO molecules, in which the two central NO molecules stretch out-of-phase. An example of these vibrations is shown in Figure 3, which compares the experimental spectrum with that simulated for the lowest energy doublet state [(NO)_6_]^+^ structure which is calculated to lie 0.08 eV above the lowest energy structure identified. This cluster typifies the challenge these structures, with their many local minima of comparable energy, present to quantum chemistry. In the case of [(NO)_6_]^+^, we calculate at least seven distinct cluster structures across three different low-lying electronic states (doublet, quartet and sextet multiplicities), all of which lie within 0.1 eV of our putative global minimum structure—see Figure S7 for details and simulated structures. At this level of theory, we consider all of these structures to be isoenergetic. Unsurprisingly, then, it proves impossible to assign the experimental spectra unambiguously to a unique cluster structure. However, structural motifs based on “core” and “solvent” NO molecules and their associated normal modes, appear to describe the experimental spectrum adequately. All low-lying structures exhibit two main (higher intensity) bands in the region of 1800 cm^–1^. Their separation, however, and their relative intensities vary between calculated structures. One mode (at 1836 cm^–1^) is a near pure local mode, centered on a terminal NO molecule bound to the double-dimer (NO)_4_^+^ core. This is particularly sensitive to small perturbations in the structure.

Although some basic structural motifs can be understood by comparing experimental and simulation spectra, it is far from clear which NO moiety is lost during the fragmentation process. By the very nature of the experiment - in that spectra are generated by monitoring the loss of an NO molecule from the homogeneous cluster—fragmentation dynamics are challenging to determine experimentally. Naively, one might expect peripheral NO molecules to be lost but internal rearrangement and the presence of smaller substructures (i.e., NO dimers and trimers) may complicate this. Indeed, many cluster sizes exhibit photofragmentation corresponding to the loss of multiple NO molecules (see Figure S1, Supporting Information).

**Figure 4:**
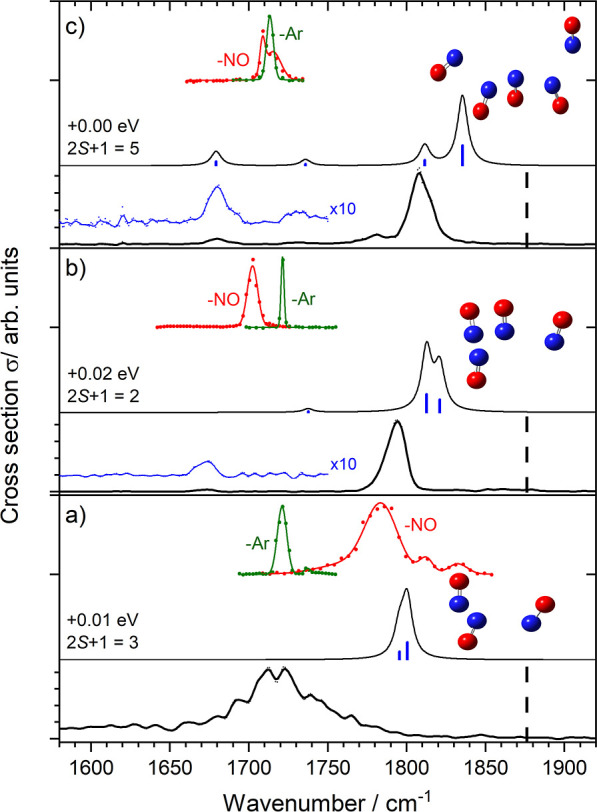
Comparison of experimental, simulated and literature (red, green)^15,20^ action spectra of (a) [(NO)_3_]^+^, (b) [(NO)_4_]^+^ and (c) [(NO)_5_]^+^ clusters. In each case, simulated IR-PD spectra are generated from representative low-lying structures from CAM-B3LYP/aug-cc-pVTZ calculations. Previous spectra are presented in both NO (red) and Ar (green) loss channels and adapted with permission from ref (15) (©1999, AIP) and ref (20) (©2005, RSC Publishing) The dashed lines mark the free NO fundamental band at 1876 cm^–1^.

It is interesting to compare the spectra in Figure 2 with those reported by Stace and co-workers for the same and Ar-tagged complexes, albeit formed in a very different cluster source. In the Stace source, cationic clusters are formed by electron impact ionization (EI) of neutral complexes and then cool by evaporation prior to infrared photodissociation.^15,20^Figure 4 shows the comparison directly for [(NO)_*n*_]^+^ (*n* = 3–5) and shows marked differences in the spectra recorded in the two studies. The spectra from the current study are shown in black, those from Stace and co-workers in red (NO loss from [(NO)_*n*_]^+^ clusters) and green (Ar loss from [Ar·(NO)_*n*_]^+^). The latter are assumed to be significantly colder given the Ar tag.

As discussed above, our [(NO)_3_]^+^ band, though broadened due to multiple photon absorption, appears at the same wavenumber as the earlier [Ar·(NO)_3_]^+^ study.^15^ The fluence of the CO laser employed in the latter was too low to drive multiple photon absorption but the charged clusters produced by EI cooled only by evaporation. The spectrum of the nontagged species from Mouhandes et al., as well as the bands in the simulated spectrum lie nearly 100 cm^–1^ to the blue of our band, although some energetically low-lying isomers (including the global minimum structure) do have bands near 1720 cm^–1^ (see Figure S4). It is possible that the DFT methods employed do not fully capture the charge delocalization in this case.

The differences between our spectra and those from the earlier studies are more marked for other clusters (Figure 4b,c). We observe the main infrared bands close to 1800 cm^–1^, some 70–100 cm^–1^ to the blue of the bands observed by Mouhandes and Stace. We attribute these differences to the contrasting sources employed. In our case, clustering starts with NO^+^ which accretes additional molecules in the throat of a supersonic expansion. By contrast, in the Stace instrument neutral (NO)_*n*_ clusters were produced first and subsequently ionized by electron impact (at 70 eV). Although ionization and subsequent fragmentation provides a mechanism for cooling the charged clusters produced, the initial structures produced are those accessible in the Franck–Condon region from low-lying structures on the neutral potential energy surface. These structures, and their IR spectra, may be significantly different from the low-lying structures of the cation. The presence of the Ar tag in the Mouhandes study, as well as providing for efficient cooling/stabilization during the ionization process, also offers the possibility of charge transfer from Ar^+^ and/or autoionization of Ar*.^56^

In order to test the possibility that the current study and that of Stace and co-workers have investigated different regions of the cation cluster potential energy surface, we have undertaken extensive computational searches. Utilizing the *Kick*^3^ algorithm, 200 initial guesses for each combination of assemblies allows sufficient sampling of the cation surface to locate a number of local minima with structures close to those of the neutral clusters. We also generated structures directly from initial guesses on the neutral surface for *n* = 3, 4. That is, we first optimized neutral cluster geometries (Figure S11), and then reoptimized the structures following removal of an electron (Figure S12). This led to several new structures, several of which do indeed exhibit vibrational bands in the 1700 cm^–1^ region (Figure S12).

For *n* = 3, this process yields some cation structures within 0.1 eV of the lowest energy structure whose spectra are dominated by bands around 1800 cm^–1^. However, other slightly higher energy structures (from +0.17 eV and above) do show bands around 1700 cm^–1^ which are in closer agreement with the spectra by Mouhandes et al.

In the case of *n* = 4, optimization of low-lying neutral structures on the cation surface does not lead to low-lying isomers (the lowest found is 0.29 eV above the global minimum). The (NO)_4_^+^ surface, though exhibits a huge number of distinct local minima (Figure S13) depending on whether optimization starts with anti/para (NO)_2_ dimer structures. It is noticeable that tetramer structures, formed from pairs of dimers, exhibit spectra with a strong absorption at 1640–1700 cm^–1^.

In short, although the calculations prove inconclusive given the complexity of the potential energy surfaces involved, it seems likely given the different cluster production methods that this study interrogates different structural forms than the studies by Stace and co-workers.

### 3.2 Infrared spectra of [NO_2_(NO)_*n*_]^+^ clusters

**Figure 5:**
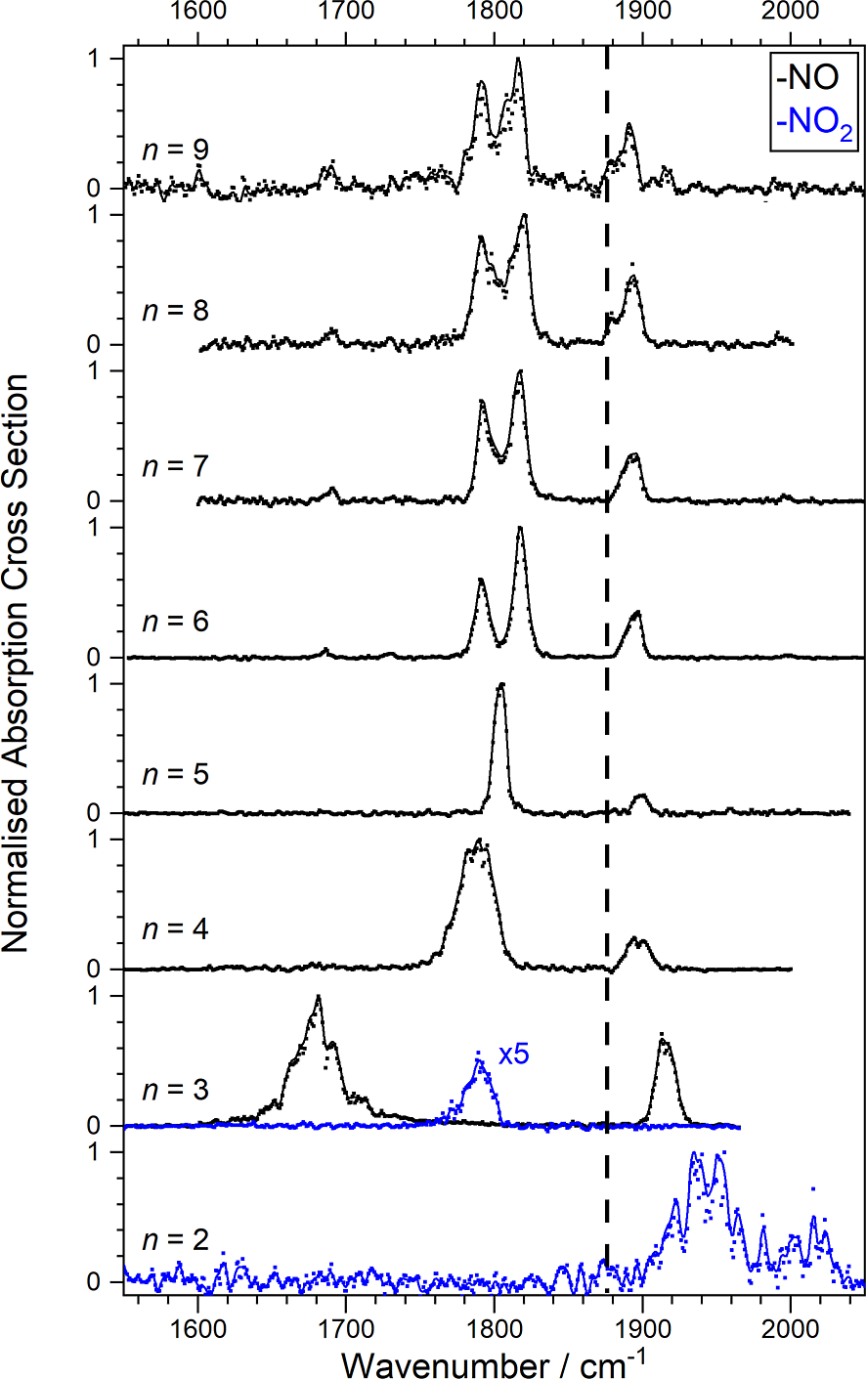
Infrared photodissociation spectra of the [NO_2_(NO)_*n*_]^+^ clusters for *n* = 2–9. Each spectrum is measured in either the NO (black) or NO_2_ (blue) loss channel (or both for *n* = 3) and has had an adjacent averaging with a window of 10 cm^–1^. The dashed line marks the fundamental band in free NO at 1876 cm^–1^.

[NO_2_(NO)_*n*_]^+^ are the second most abundant species identified in the mass spectrum. The IR-PD spectra of the [NO_2_(NO)_*n*_]^+^ (*n* = 2–9) species are presented in Figure 5. The assignment of [NO_2_(NO)_*n*_]^+^ is based on the fact that the smallest members of this series, *n* = 2, 3, exhibit IR-induced NO_2_ loss as shown, suggesting that NO_2_ is formed during the clustering. This assignment is supported by the example simulated spectra shown in Figures S15 (Supporting Information).

A noticeable difference in all the spectra shown in Figure 5 compared to those for [(NO)_*n*_]^+^ (Figure 2) is the new strong band observed around 1900 cm^–1^. This band does not, as might be expected, reflect vibrations in the NO_2_^(+)^ moiety but rather from NO/(NO)_2_ vibrations whose oscillator strength is enhanced by proximity to the NO_2_. In [NO_2_(NO)_2_]^+^ and [NO_2_(NO)_3_]^+^ this is a blue-shifted NO stretch in a [NO_2_–NO] dimer. For [NO_2_(NO)_*n*_]^+^ (*n* ≥ 4) the NO_2_ moiety is solvated by a pair of NO dimers each of which exhibits in- and out-of-phase NO stretches at 1940 and 1800 cm^–1^, respectively as previously observed in metal nitrosyl complexes.^34^

**Figure 6:**
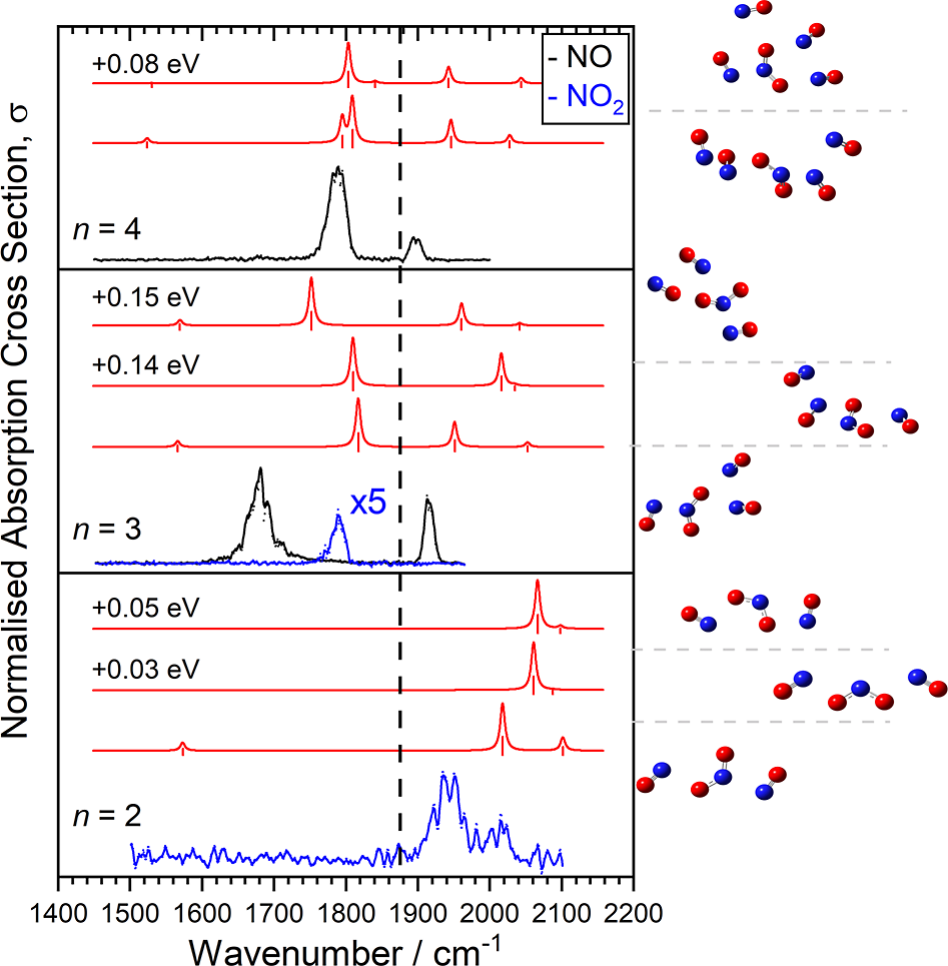
Comparison of experimental and a representative simulated infrared action spectra of the [NO_2_(NO)_2–4_]^+^ clusters. Simulated spectra for a number of low-lying structures are included (red) with their corresponding relative energies denoted against each trace. Corresponding cluster geometries for each simulated spectrum is shown on the right. The dashed line marks the fundamental band in free NO at 1876 cm^–1^.

Unlike in the pure [(NO)_*n*_]^+^ complexes, in which the charge is highly delocalized across the whole complex with the outer NO carrying slightly more positive charge, [NO_2_(NO)_*n*_]^+^ exhibit significant electron donation from NO to NO_2_ leading to a small negative charge on the oxygen atoms of the latter. This is despite the similarity of the NO and NO_2_ ionization energies (9.264 and 9.56 eV, respectively) both of which are higher than the IP of (NO)_*n*_ (8.75 eV).^6,12^ The net effect is stronger binding of the NO^δ+^ ligands leading to a blue-shift in the observed vibration. In all low-energy calculated structures, the NO_2_ moiety lies at the core of the complex and is solvated by NO molecules (See, for example, Figure 6).

The apparently embedded nature of the NO_2_ moiety makes the observation of NO_2_ loss from the [NO_2_(NO)_2_]^+^ complex intriguing. The lowest energy structure we find containing an identifiable NO dimer lies 0.25 eV above the putative global minimum. NO_2_ loss from such a cluster might be expected if the charge resides preferentially on the NO dimer. However, the spectrum of this complex is not consistent with the NO_2_ loss spectrum and is not shown in Figure 6. We have considered the possibility of O atom transfer reaction but this seems unlikely on the ground state ON-ONO singlet surface (see Figure S9). On the triplet surface, however, there is a discontinuity at 1.4 Å associated with rearrangements to NO_2_ bound out of plane to an *anti*-NO dimer.

The spectra of the [NO_2_(NO)_3_]^+^ complex shown in Figures 5 and 6 are markedly different in the NO loss and NO_2_ loss channels, the most likely explanation for which is the coexistence of two different structures with one exhibiting a terminal NO_2_. The NO-loss spectrum has one broad band around 1680 cm^–1^ reminiscent of the (NO)_3_^+^ spectrum plus a band around 1920 cm^–1^ which can be assigned to the in-phase dimer stretch as above. The NO_2_ loss spectrum comprises a weaker single feature around 1800 cm^–1^ which, by comparison with other experimental and calculated spectra, is assigned as an out-of-phase NO-dimer stretch. Figure 6 shows the comparison with simulated spectra. The simulated spectra all lie somewhat to the blue of the experimental spectra but otherwise reproduce the main features (number and spectral separation of bands). Additional calculated low-energy structures are given in Figure S15.

For the [NO_2_(NO)_4_]^+^ clusters the calculated spectra exhibit similar vibrational motifs as for the *n* = 3 clusters, but provide more convincing agreement with the experimental spectrum. The only fragmentation channel observed for this and larger clusters is the NO loss channel suggesting complete embedding of the NO_2_. The bands around 1800 and 1900 cm^–1^ are common to all clusters *n* ≥ 4, with additional splitting in the 1800 cm^–1^ band beyond *n* = 5 as multiple NO dimer formation (cis- and anti-) becomes possible. The splitting is accompanied by a weak satellite peak near 1700 cm^–1^ which may be the NO_2_ asymmetric stretch in the largest clusters calculated (found at 1616 cm^–1^ in the free molecule)^52^ but could also be dimer stretches as assigned in metal-nitrosyl ion–molecule complexes.^34,57^

### 3.3 Intracluster chemistry in [(NO)_*n*_]^+^ and [NO_2_(NO)_*n*_]^+^ clusters

Finally, in addition to photofragmentation, we consider the evidence of intracluster chemistry within or during the formation of [(NO)_*n*_]^+^ and [NO_2_(NO)_*n*_]^+^ clusters. For *n* ≥ 7 in both the pure NO and oxidized clusters IR-PD spectra exhibit fragmentation patterns that are indicative of both N_2_O (nitrous oxide) as well as NO loss. IR-PD spectra for both NO and N_2_O loss channels for [(NO)_7,8_]^+^ and [NO_2_(NO)_8,9_]^+^ are provided in Figure S3 in Supporting Information. N_2_O loss suggests the intracluster formation of [(NO_2_)(N_2_O)] moieties with the closed-shell N_2_O likely the more weakly bound within the cluster. Indeed, N_2_O loss from [(NO_2_)(N_2_O)(NO)_*n*_]^+^ clusters may well be the origin of the [(NO_2_)(NO)_*n*_]^+^ clusters considered above.

**Figure 7:**
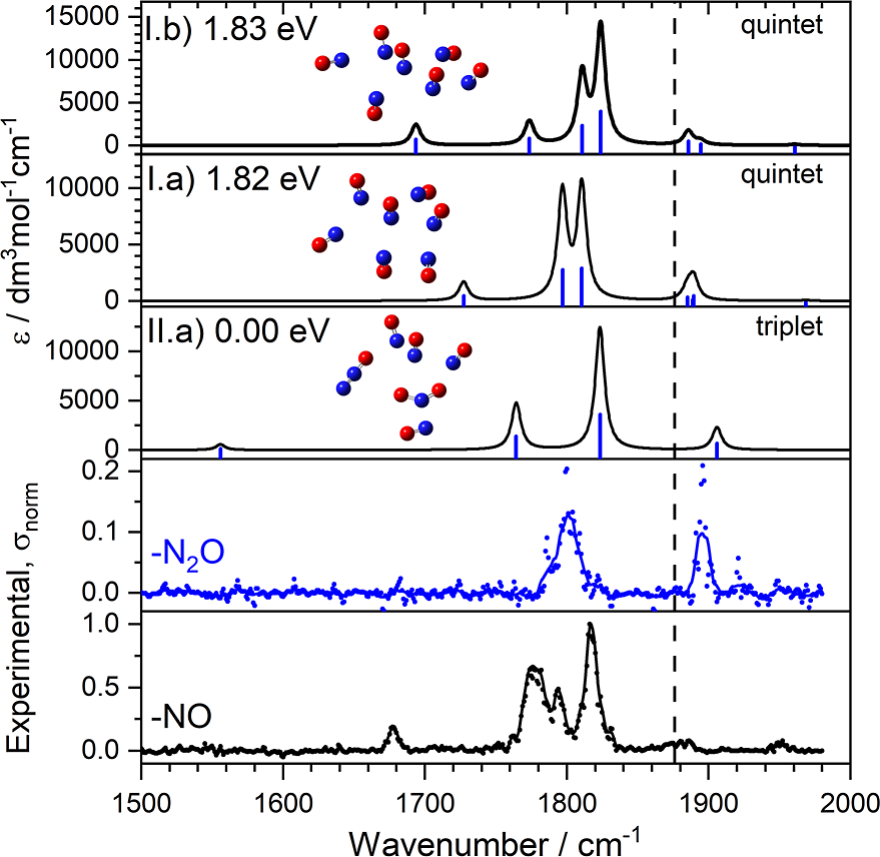
Comparison of experimental and a representative simulated infrared action spectra of the [(NO)_7_]^+^ cluster illustrating both NO (black) and N_2_O (blue) fragmentation. Simulated spectra for a number of structures are included noting that the pure [(NO)_7_]^+^ geometries lie significantly higher in energy than the [(NO_2_)(N_2_O)(NO)_4_]^+^ cluster. The dashed line marks the fundamental band in free NO at 1876 cm^–1^.

Figure 7 compares the experimental spectra of [(NO)_7_]^+^ observed in both fragmentation channels with those simulated for calculated structures of both homogeneous [(NO)_7_]^+^ and [(NO_2_)(N_2_O)(NO)_4_]^+^ clusters. The latter are calculated to lie 1.8 eV below the homogeneous clusters albeit behind a considerable kinetic barrier given the bond-breaking necessarily involved in its formation. The different spectra observed in the two fragmentation channels is indicative of two different structures, rather than differing fragmentation efficiencies (fragment quantum yields) of the same structure, whose spectrum would be similar in all channels. As discussed above the spectrum in the NO loss channel is dominated by NO-based vibrations around 1800 cm^–1^ which are reasonably well reproduced in the calculations (structures I.a and I.b) in Figure 7, the lowest energy calculated structures of [(NO)_7_]^+^, but representative of many of very similar energy. The simulations also capture the weaker band to the red of the main peaks that is associated with an out-of-phase stretching mode of 4 central NO molecules (as depicted in Figure 3).

The spectrum recorded in the N_2_O loss channel is markedly different and comprises one (likely blended) band at 1800 cm^–1^ and a similarly strong band at 1900 cm^–1^. It bears close resemblance to the spectra of the [NO_2_(NO)_*n*_]^+^ clusters in Figure 5. The two main peaks are reproduced well in the simulated spectrum of the [(NO_2_)(N_2_O)(NO)_4_]^+^ cluster (structure II.a), but the third band, calculated to the red, appears as a shoulder to the main peak at 1800 cm^–1^. We calculate the binding energy of N_2_O to the [(NO_2_)(NO)_4_]^+^ cluster to be 0.28 eV, only slightly larger than the photon energy at these wavenumbers and consistent with single-photon dissociation. For comparison, we calculated the NO binding energy to (NO)_6_^+^ at 0.14 eV.

We have not attempted to calculate the barrier to intracluster reaction which would be a significant undertaking. Given the calculated exoergicity of the (NO)_3_^+^ → (NO_2_)(N_2_O)^+^ process (≥1.8 eV), and the spectroscopic evidence above, we believe that it is highly likely that many of the stable species observed in our mass spectrum, and hence investigated spectroscopically, result from the fragmentation of larger clusters, e.g.
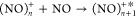
5
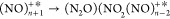
6
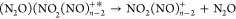
7
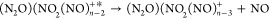
8

### 3.4 Summary and conclusions

Infrared action spectroscopy, in conjunction with quantum chemical calculations of low-energy isomers has revealed new information on the structures of size-selected (NO)_*n*_^+^ and NO_2_(NO)_*n*_^+^ clusters. Unsurprisingly for clusters of open-shell molecules, the rich potential energy landscape results in very many near isoenergetic low-lying structures. The spectra of our clusters, produced by nucleating NO molecules around an initial NO^+^ ion, differ markedly from those of the same species in the literature produced by electron impart ionization of neutral (NO)_*n*_ complexes. Our calculations suggest that, in the literature studies, ionization in the Franck–Condon region likely produces cationic structures reminiscent of the neutral cluster geometries.

We observe clear evidence of bond-breaking/making chemistry within (NO)_*n*_^+^ clusters in the fact that some species exhibit very different spectra in different fragmentation channels. We interpret these changes as resulting from formation of mixed nitrous oxide/nitrogen dioxide/nitric oxide complexes, (N_2_O)(NO_2_)(NO)_*n*_^+^, despite the likely barriers involved in such intracluster reactions. Such species are indistinguishable by mass from (NO)_*n*+3_^+^ clusters and can only be identified spectroscopically.

## Supporting Information Available

Additional spectra, example fragmentation mass spectra; dissociation branching ratios (PDF)
