# Supplementary material for: Infrared Photodissociation Spectroscopy of Cationic Nitric Oxide Clusters, [(NO)n]+, and [NO2(NO)n]+
Source: J Phys Chem A. 2025 Apr 21;129(17):3867–75. doi: 10.1021/acs.jpca.5c01377 (PMC12051196; doi:10.1021/acs.jpca.5c01377)
Supplement: Supplementary file 1 — jp5c01377_si_001.pdf [file jp5c01377_si_001.pdf]

Infrared photodissociation spectroscopy of cationic nitric oxide clusters, $[(NO)_n]^+$, and $[NO_2(NO)_n]^+$

*Peter D. Watson,[1] Gabriele Meizyte,[1] Philip A. J. Pearcy,[1] Edward I. Brewer,[1] Alice E. Green,[1] Anthony J. Stace[2] and Stuart R. Mackenzie[1]**

[1] Department of Chemistry, University of Oxford, Chemistry Research Laboratory, Mansfield Road, Oxford, United Kingdom, OX1 3TA

[2] University of Nottingham, School of Chemistry, University Park, Nottingham, United Kingdom, NG7 2RD

Supporting Information

Contents:

Figure S1: Experimental IR-PD spectra of the $[(NO)_n]^+$ complexes for n = 3-8. IR-PD spectra are recorded in multiple NO loss channels (up to 4 NO molecules for $[(NO)_8]^+$). 4
Figure S2: Experimental IR-PD spectra of the $[NO_2(NO)_n]^+$ complexes for n = 4-9. IR-PD spectra are recorded in multiple NO loss channels. In one case for n = 9 denoted by a red asterisk, depletion is not observed in all loss channels (i.e., loss of 2 NO). 5
Figure S3: Experimental IR-PD spectra of the $[(NO)_{7,8}]^+$ and $[NO_2(NO)_{8,9}]^+$ complexes. IR-PD spectra are shown in both NO and N_2O loss channels (inferred by the remaining mass of the complex). 6
Figure S4: Comparison of experimental, literature[1] and simulated infrared action spectra of $[(NO)_3]^+$ complexes. The experimental IR-PD spectrum (black) is recorded in the loss of NO from the cluster, corresponding spectra from Stace and co-workers. are recorded in both the loss of NO (red) and Ar (green) channels. Simulated spectra are generated from CAM-B3LYP/*aug-cc*-pVTZ calculations and scaled to the free NO stretching frequency (1876 cm^{-1}, SF = 0. 9268). 7
Figure S5: Comparison of experimental, literature[1] and simulated infrared action spectra of $[(NO)_4]^+$ complexes. The experimental IR-PD spectrum (black) is recorded in the loss of NO from the cluster, corresponding spectra from Stace and co-workers are recorded in both the loss of NO (red) and Ar (green) channels. Simulated spectra are generated from CAM-B3LYP/*aug-cc*-pVTZ calculations and scaled to the free NO stretching frequency (1876 cm^{-1}, SF = 0. 9268). 8
Figure S6: Comparison of experimental, literature[1] and simulated infrared action spectra of $[(NO)_5]^+$ complexes. The experimental IR-PD spectrum (black and blue) is recorded in the loss of NO from the cluster, corresponding spectra from Stace and co-workers are recorded in both the loss of NO (red) and Ar (green) channels. Simulated spectra are generated from CAM-B3LYP/*aug-cc*-pVTZ calculations and scaled to the free NO stretching frequency (1876 cm^{-1}, SF = 0. 9268). Calculated structures are denoted with Roman numerals corresponding to the substituents of the initial guess and referred to in Figures S19-S36 (i.e., IV structures are generated from 2(*para*-$(NO)_2$) + NO). 9
Figure S7: Comparison of experimental and simulated infrared action spectra of $[(NO)_6]^+$ complexes. The experimental IR-PD spectrum (black) is recorded in the loss of NO from the cluster. Simulated spectra are generated from CAM-B3LYP/*aug-cc*-pVTZ calculations and scaled to the free NO stretching frequency (1876 cm^{-1}, SF = 0. 9268). 10
Figure S8: Comparison of experimental and simulated infrared action spectra of $[(NO)_7]^+$ complexes. The experimental IR-PD spectrum (black) is recorded in the loss of NO from the cluster. Simulated spectra are generated from CAM-B3LYP/*aug-cc*-pVTZ calculations and scaled to the free NO stretching frequency (1876 cm^{-1}, SF = 0. 9268). Calculated structures are denoted with Roman numerals corresponding to whether they are generated from $[(NO)_7]^+$ or $[(N_2O)(NO_2)(NO)_4]^+$ complexes. 11
Figure S9: Relaxed potential energy scan along the ON-ONO distance in the $[NO_2(NO)_2]^+$ complex. Relaxed scans are performed on both the singlet (black) and triplet (green) surfaces with CAM-B3LYP/*aug-cc*-pVTZ energies reported relative to the minimum singlet energy. 12
Figure S10: Simulated IR-PD spectra for the $[(NO)_3]^+$ complexes. Simulated spectra are generated from CAM-B3LYP/*aug-cc*-pVTZ calculations and scaled to the free NO stretching frequency (1876 cm^{-1}, SF = 0. 9268). 13
Figure S11: Simulated IR-PD spectra for the $[(NO)_3]$ complexes. Simulated spectra are generated from CAM-B3LYP/*aug-cc*-pVTZ calculations and scaled to the free NO stretching frequency (1876 cm^{-1}, SF = 0. 9268) and convoluted with a Lorentz function (FWHM = 8 cm^{-1}). 14
Figure S12: Simulated IR-PD spectra for the *ionised* $[(NO)_3]$ complexes. Simulated spectra are generated from CAM-B3LYP/*aug-cc*-pVTZ calculations of the cation complexes from optimised neutral geometries as starting

guesses. Vibrational frequencies are scaled to the free NO stretching frequency (1876 cm^{-1}, SF = 0. 9268) and convoluted with a Lorentz function (FWHM = 8 cm^{-1}). Energies are given relative to the lowest energy $(NO)_n^+$ structure identified. 15
Figure S13: Simulated IR-PD spectra for the $[(NO)_4]^+$ complexes. Simulated spectra are generated from CAM-B3LYP/aug-cc-pVTZ calculations and scaled to the free NO stretching frequency (1876 cm^{-1}, SF = 0. 9268). 16
Figure S14: Simulated IR-PD spectra for the $[(NO)_5]^+$ complexes from 2(*anti*-$(NO)_2$) + NO. Simulated spectra are generated from CAM-B3LYP/*aug-cc*-pVTZ calculations and scaled to the free NO stretching frequency (1876 cm^{-1}, SF = 0. 9268). 17
Figure S15: Simulated IR-PD spectra for the $[NO_2(NO)_3]^+$ complexes. Simulated spectra are generated from CAM-B3LYP/aug-cc-pVTZ calculations and scaled independently to the free NO stretching frequency (1876 cm^{-1}, SF = 0. 9268) and NO_2 asymmetric stretch (1616 cm^{-1}, SF = 0.9252). 18

A. Additional Experimental Action Spectra:

Figures S1-S3 present a series of additional experimental spectra that illustrate multiple and/or interested fragmentation pathways and their corresponding IRPD spectra. While many IRPD spectra are scaled to illustrate spectral features we see repeatedly the loss of features at lower masses as structural motifs are no longer preserved (i.e. absence of peaks ~1700cm^{-1} for masses less than or equal to 120 amu in the pure $[(NO)_n]^+$ clusters).

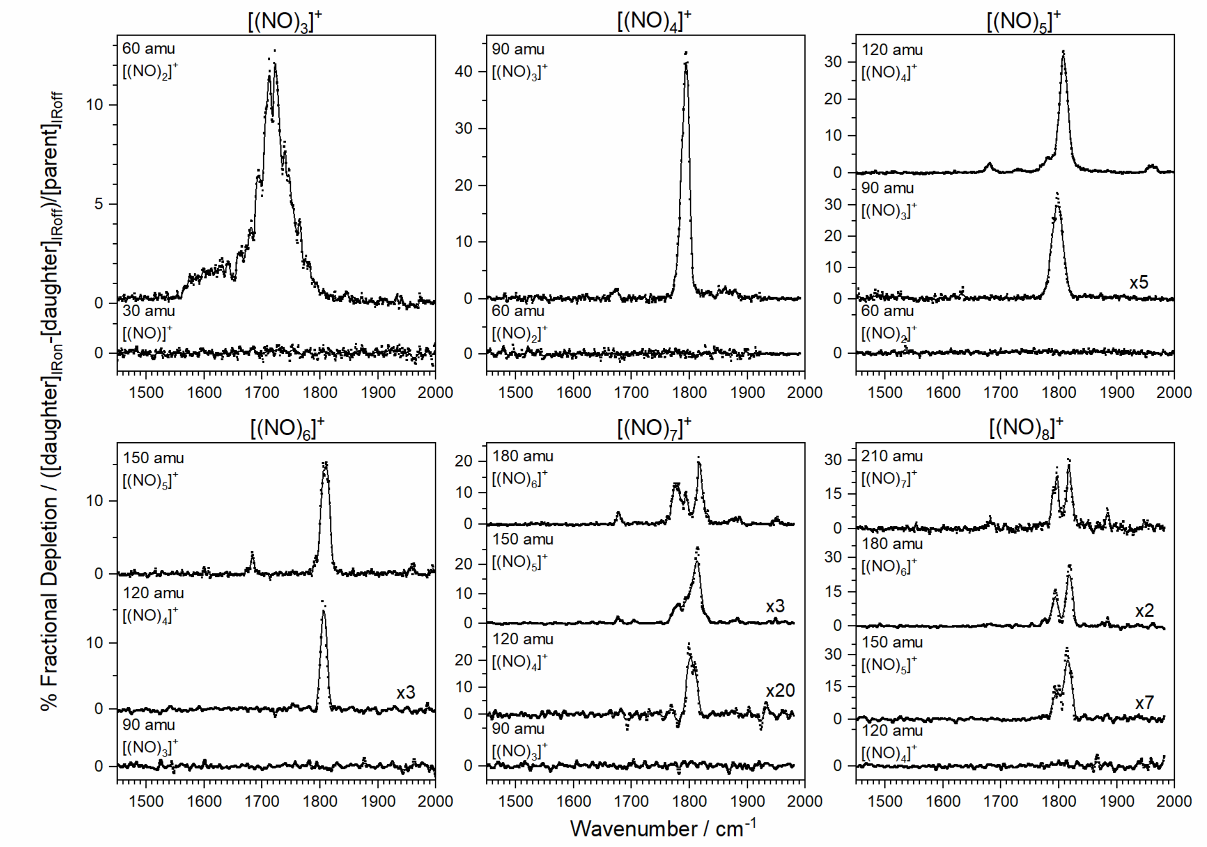

Figure S1: Experimental IR-PD spectra of the $[(NO)_n]^+$ complexes for n = 3-8. IR-PD spectra are recorded in multiple NO loss channels (up to 4 NO molecules for $[(NO)_8]^+$).

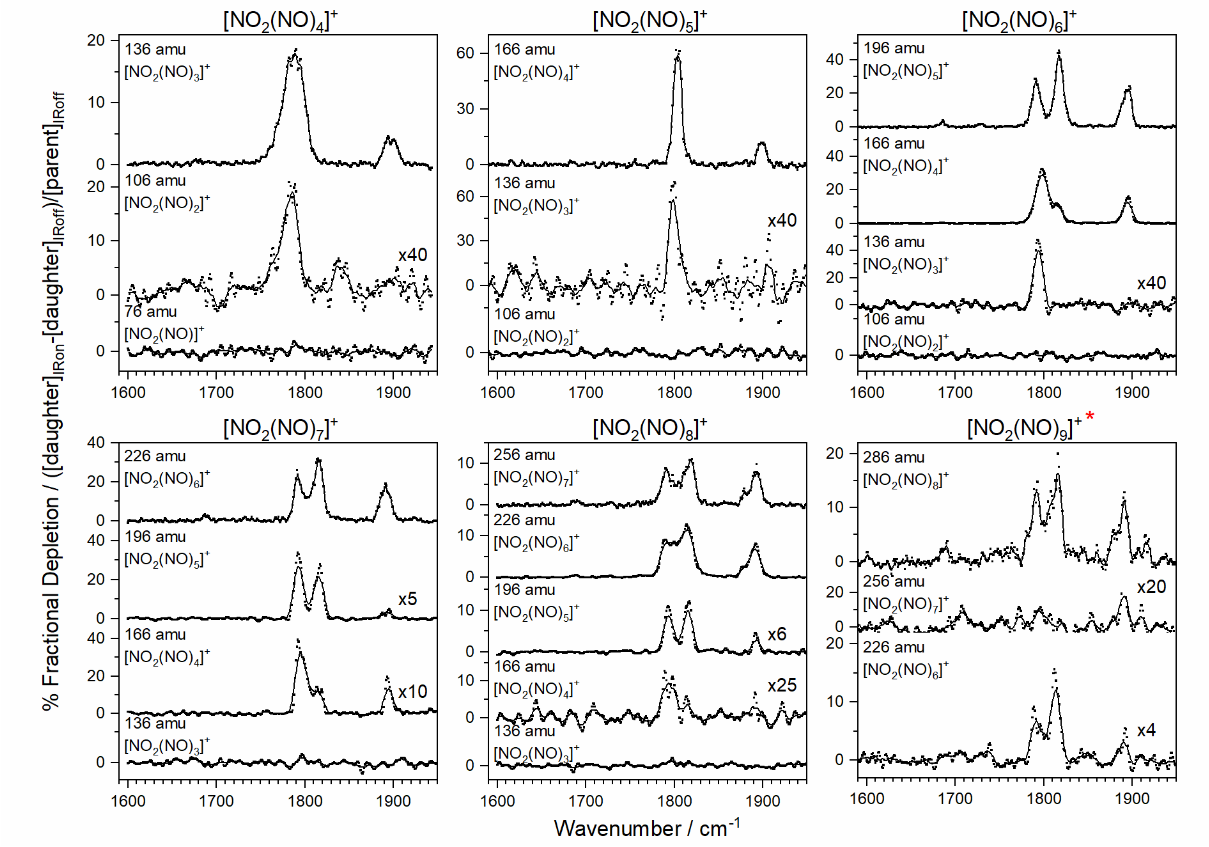

Figure S2: Experimental IR-PD spectra of the $[NO_2(NO)_n]^+$ complexes for n = 4-9. IR-PD spectra are recorded in multiple NO loss channels. In one case for n = 9 denoted by a red asterisk, depletion is not observed in all loss channels (i.e., loss of 2 NO).

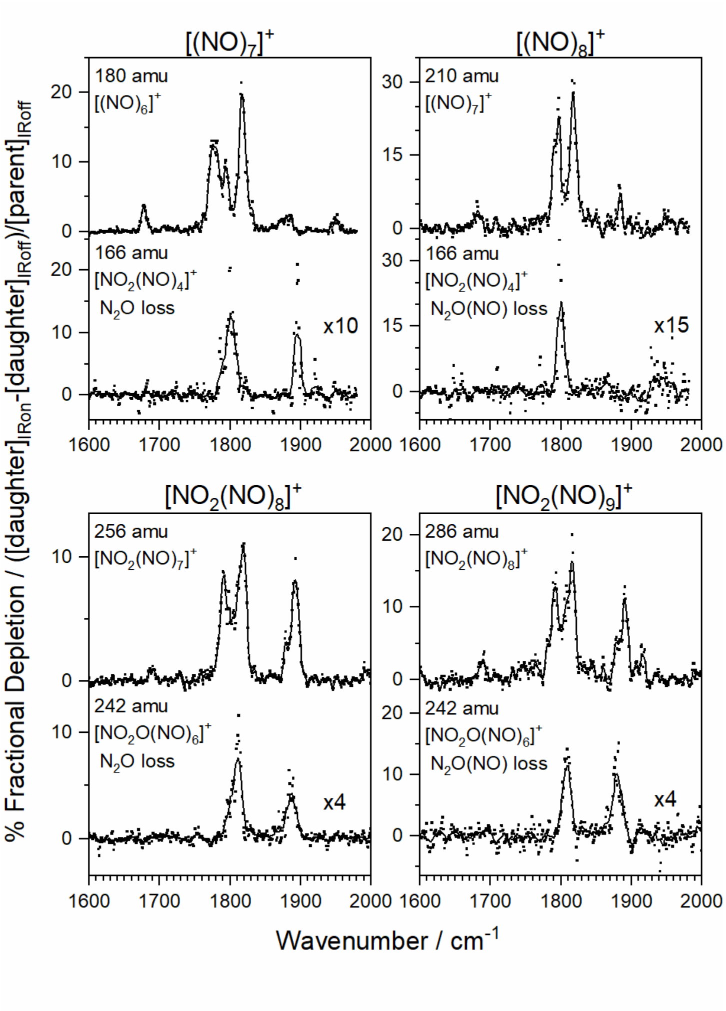

Figure S3: Experimental IR-PD spectra of the $[(NO)_{7,8}]^+$ and $[NO_2(NO)_{8,9}]^+$ complexes. IR-PD spectra are shown in both NO and N_2O loss channels (inferred by the remaining mass of the complex).

B. Comparison Between Experimental, Literature and Simulated Spectra of $[(NO)_n^+]$:

To assist in the assignment of experimental spectra, a range of simulated spectra are compared with both the experimental spectra recorded as part of this work and those in literature from Stace et al.[1, 2] (Figures S4-S8). For $[(NO)_{3-5}]^+$, higher energy cluster structures are included in simulated spectra to illustrate agreement with Stace et al. where electron impact ionization of neutral $(NO)_n$ is believed to yield local minima above the global minimum on the cation potential energy surface.

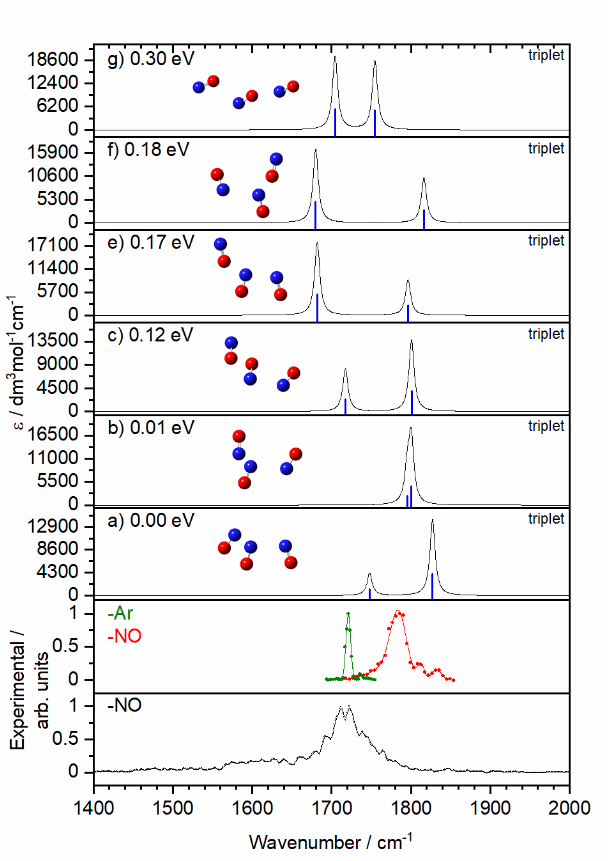

Figure S4: Comparison of experimental, literature[1, 2] and simulated infrared action spectra of $[(NO)_3]^+$ complexes. The experimental IR-PD spectrum (black) is recorded in the loss of NO from the cluster, corresponding spectra from Stace and co-workers. are recorded in both the loss of NO (red) and Ar (green) channels. Simulated spectra are generated from CAM-B3LYP/*aug-cc*-pVTZ calculations and scaled to the free NO stretching frequency (1876 cm^{-1}, SF = 0. 9268).

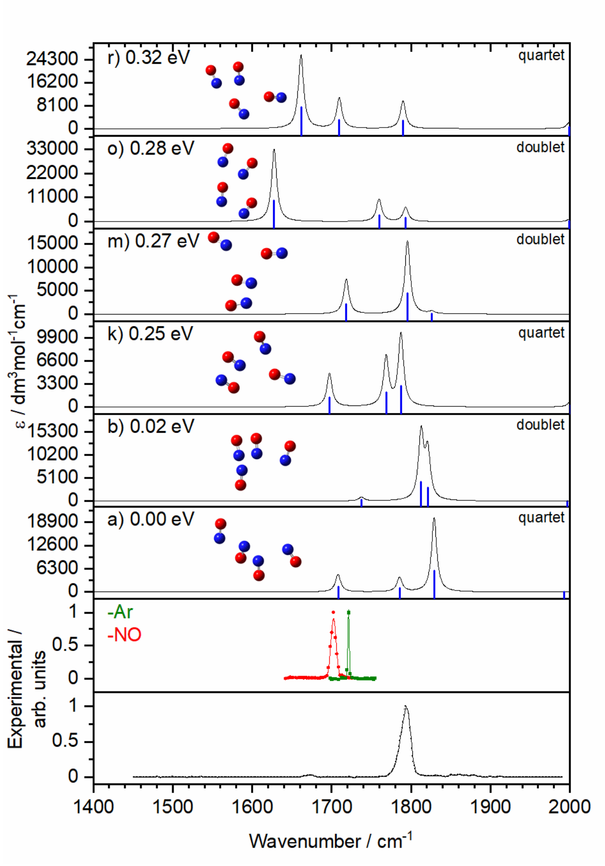

Figure S5: Comparison of experimental, literature[1, 2] and simulated infrared action spectra of $[(NO)_4]^+$ complexes. The experimental IR-PD spectrum (black) is recorded in the loss of NO from the cluster, corresponding spectra from Stace and co-workers are recorded in both the loss of NO (red) and Ar (green) channels. Simulated spectra are generated from CAM-B3LYP/*aug-cc*-pVTZ calculations and scaled to the free NO stretching frequency (1876 cm^{-1}, SF = 0. 9268).

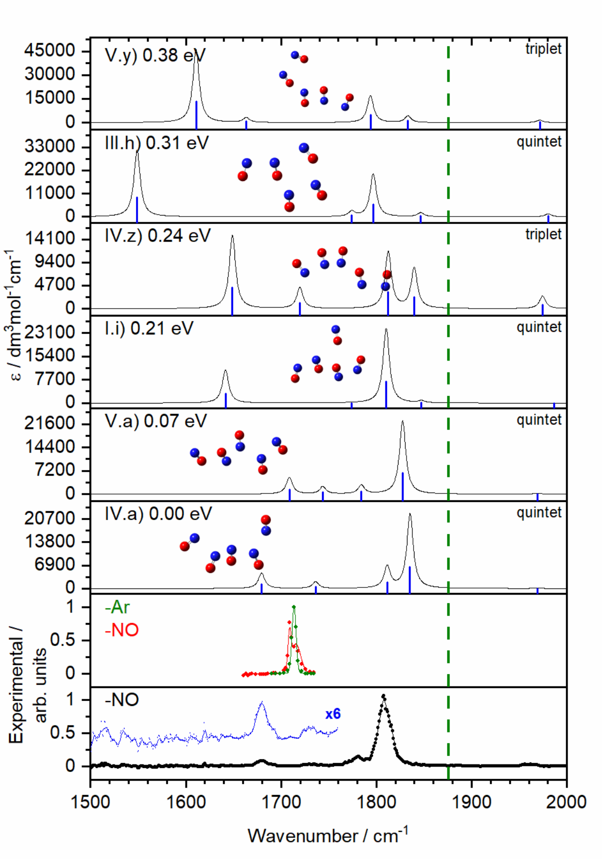

Figure S6: Comparison of experimental, literature[1, 2] and simulated infrared action spectra of $[(NO)_5]^+$ complexes. The experimental IR-PD spectrum (black and blue) is recorded in the loss of NO from the cluster, corresponding spectra from Stace and co-workers are recorded in both the loss of NO (red) and Ar (green) channels. Simulated spectra are generated from CAM-B3LYP/*aug-cc*-pVTZ calculations and scaled to the free NO stretching frequency (1876 cm^{-1}, SF = 0. 9268). Calculated structures are denoted with Roman numerals corresponding to the substituents of the initial guess and referred to in Figures S19-S36 (i.e., IV structures are generated from 2(*para*-$(NO)_2$) + NO).

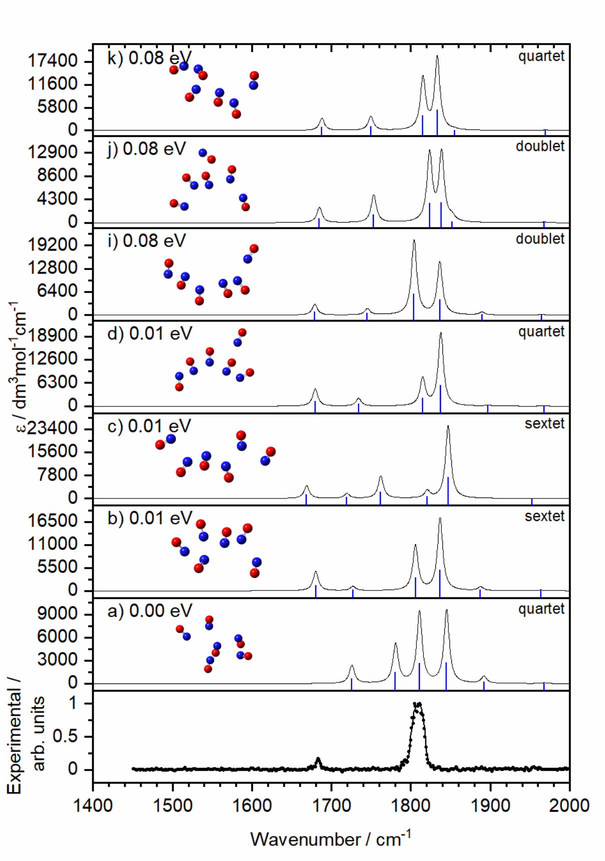

Figure S7: Comparison of experimental and simulated infrared action spectra of $[(NO)_6]^+$ complexes. The experimental IR-PD spectrum (black) is recorded in the loss of NO from the cluster. Simulated spectra are generated from CAM-B3LYP/*aug*-*cc*-pVTZ calculations and scaled to the free NO stretching frequency (1876 cm^{-1}, SF = 0. 9268).

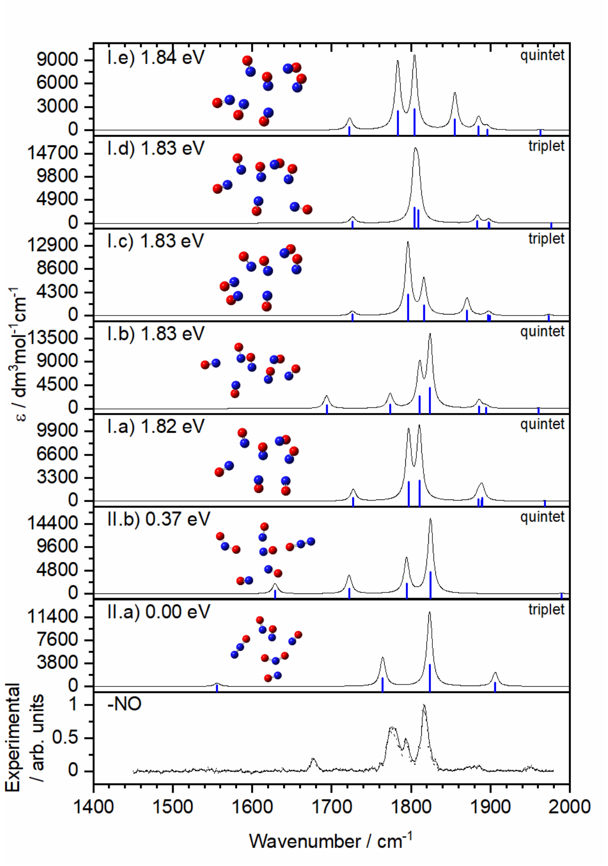

Figure S8: Comparison of experimental and simulated infrared action spectra of $[(NO)_7]^+$ complexes. The experimental IR-PD spectrum (black) is recorded in the loss of NO from the cluster. Simulated spectra are generated from CAM-B3LYP/*aug-cc*-pVTZ calculations and scaled to the free NO stretching frequency (1876 cm^{-1}, SF = 0. 9268). Calculated structures are denoted with Roman numerals corresponding to whether they are generated from $[(NO)_7]^+$ or $[(N_2O)(NO_2)(NO)_4]^+$ complexes.

C. Computational Details:

In addition to the calculations and simulated spectra presented in the main text. We include here additional computational data. Figure S9 includes a preliminary relaxed 1D scan of the O-atom transfer in the $[NO_2(NO)_2]^+$ cluster on both singlet and triplet surfaces. We note the discontinuity at ~1.4 Å on the triplet surface and suggest that internal conversion of NO_2 in such complexes will likely proceed via an intersystem crossing.

The following Figures S10-S12 complement Figure S4 and support discussion in the main text distinguishing between cationic clusters formed via laser vaporization followed by solvation (this work) and solvation followed by electron impact ionization (Stace *et al*.). Figure S10 presents the lowest optimised cationic cluster structures representative of a laser vaporization and solvation clustering process. Figure S11 presents the geometries and infrared spectra of neutral $(NO)_3$ clusters and then Figure S12 illustrates how these spectra change upon vertical excitation to the cation surface followed by relaxation to local minima. In Figure S12, the energy ordering of Figure S11 is retained and thereby appears disordered.

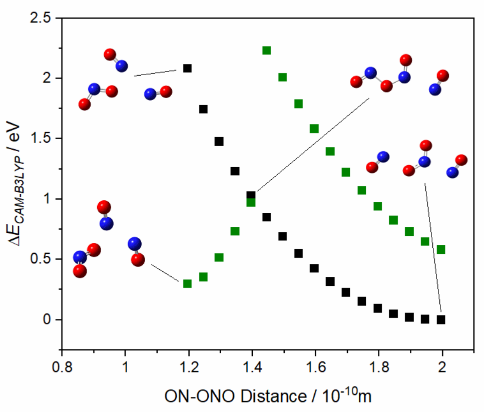

Figure S9: Relaxed potential energy scan along the ON-ONO distance in the $[NO_2(NO)_2]^+$ complex. Relaxed scans are performed on both the singlet (black) and triplet (green) surfaces with CAM-B3LYP/*aug*-*cc*-pVTZ energies reported relative to the minimum singlet energy.

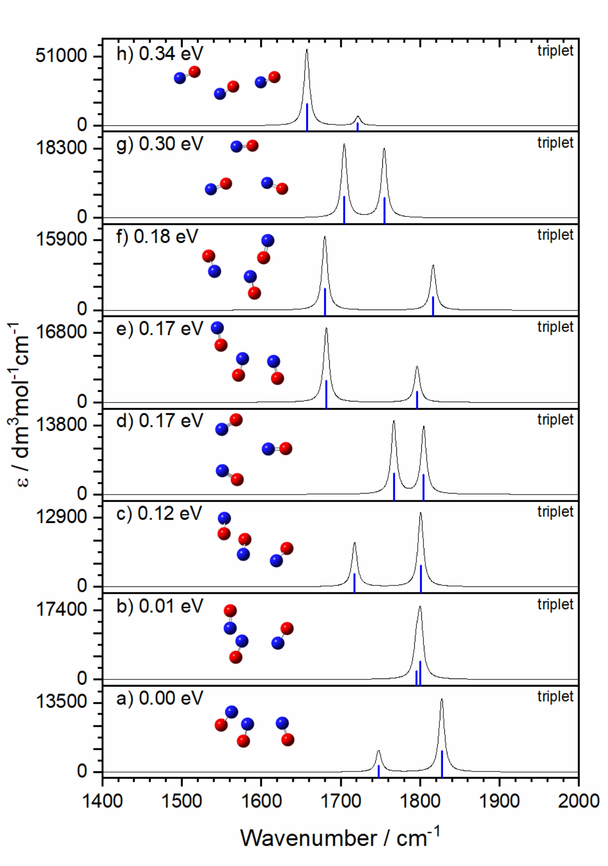

Figure S10: Simulated IR-PD spectra for the $[(NO)_3]^+$ complexes. Simulated spectra are generated from CAM-B3LYP/*aug-cc*-pVTZ calculations and scaled to the free NO stretching frequency (1876 cm^{-1}, SF = 0. 9268).

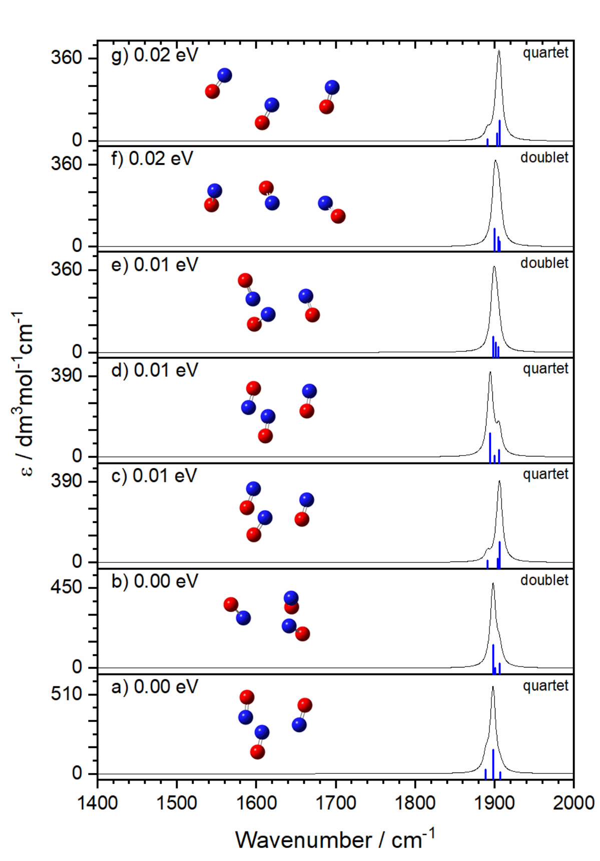

Figure S11: Simulated IR-PD spectra for the $[(NO)_3]$ complexes. Simulated spectra are generated from CAM-B3LYP/*aug*-*cc*-pVTZ calculations and scaled to the free NO stretching frequency (1876 cm^{-1}, SF = 0. 9268) and convoluted with a Lorentz function (FWHM = 8 cm^{-1}).

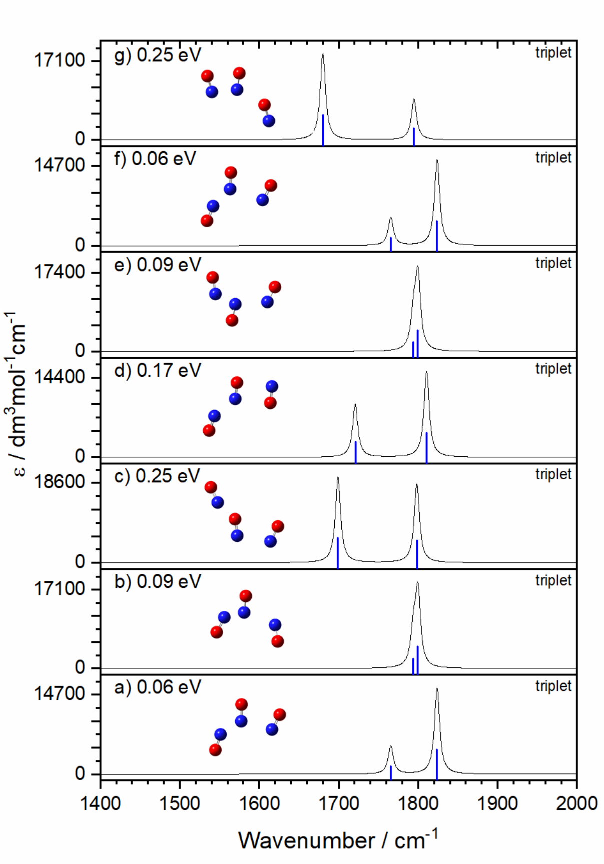

Figure S12: Simulated IR-PD spectra for the *ionised* [$(NO)_3$] complexes. Simulated spectra are generated from CAM-B3LYP/*aug-cc*-pVTZ calculations of the cation complexes from optimised neutral geometries as starting guesses. Vibrational frequencies are scaled to the free NO stretching frequency (1876 cm^{-1}, SF = 0. 9268) and convoluted with a Lorentz function (FWHM = 8 cm^{-1}). Energies are given relative to the lowest energy $(NO)_n^+$ structure identified.

The remaining Figures include simulated spectra for $[(NO)_n]^+$ and $[NO_2(NO)_n]^+$ clusters. Figure S13 includes spectra of the $[(NO)_4]^+$ cluster generated on the cation surface. These clusters were treated similarly to the $[(NO)_3]^+$ clusters with additional structures and predicted spectra from neutral $(NO)_3$ and the corresponding ionized cationic clusters also calculated and available upon request. Figure S13 shows the conformational complexity present with the lowest 20 structures all lying within 0.35 eV of the putative global minimum.

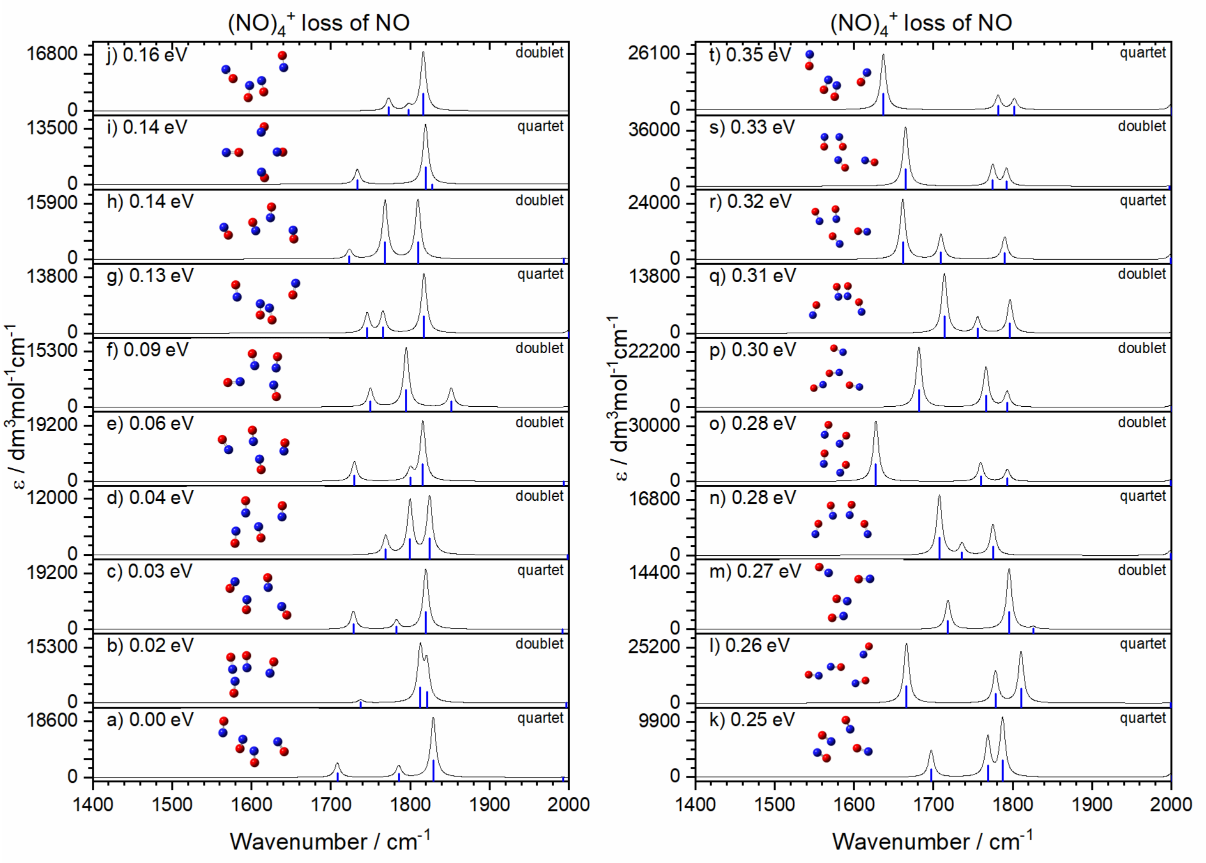

Figure S13: Simulated IR-PD spectra for the $[(NO)_4]^+$ complexes. Simulated spectra are generated from CAM-B3LYP/aug-cc-pVTZ calculations and scaled to the free NO stretching frequency (1876 cm^{-1}, SF = 0. 9268).

Similarly, for the $n = 5$ clusters the number of minima is extensive. Figure S14 shows the cationic clusters generated from two *anti*-$(NO)_2$ dimers and a NO molecule (denoted I). The lowest energy conformer here still lies 0.14 eV above the putative global minima. Spectra from other conformers are not included here but available upon request, noting that for $n = 5$; $(NO)_4$ + NO is denoted II, 5(NO) is denoted III, two *para*-$(NO)_2$ dimers and an NO is denoted IV, and a *para*-$(NO)_2$ dimer with an *anti*-$(NO)_2$ dimer and an NO molecule is denoted V. Additional predicted spectra for n = 6, 7 is also available upon request.

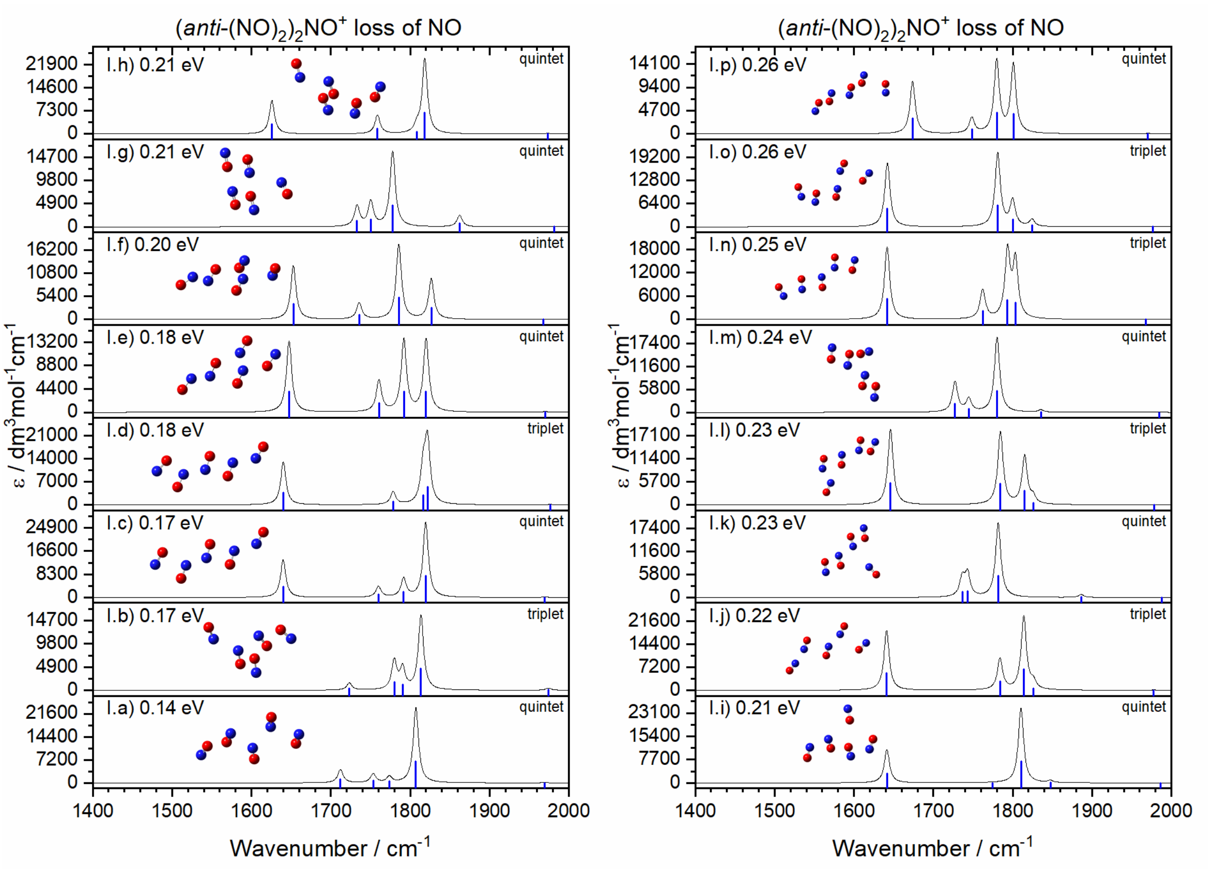

Figure S14: Simulated IR-PD spectra for the $[(NO)_5]^+$ complexes from 2(*anti*-$(NO)_2$) + NO. Simulated spectra are generated from CAM-B3LYP/*aug*-*cc*-pVTZ calculations and scaled to the free NO stretching frequency (1876 cm^{-1}, SF = 0. 9268).

Simulated $[NO_2(NO)_n]^+$ spectra

Finally, we include simulated spectra for the $[NO_2(NO)_n]^+$ spectra. Figure S15 shows the simulated spectra for n = 3 for the lowest 16 structures (all within 0.50 eV). Spectra are also generated for n = 2, 4 and 5 and is available upon request.

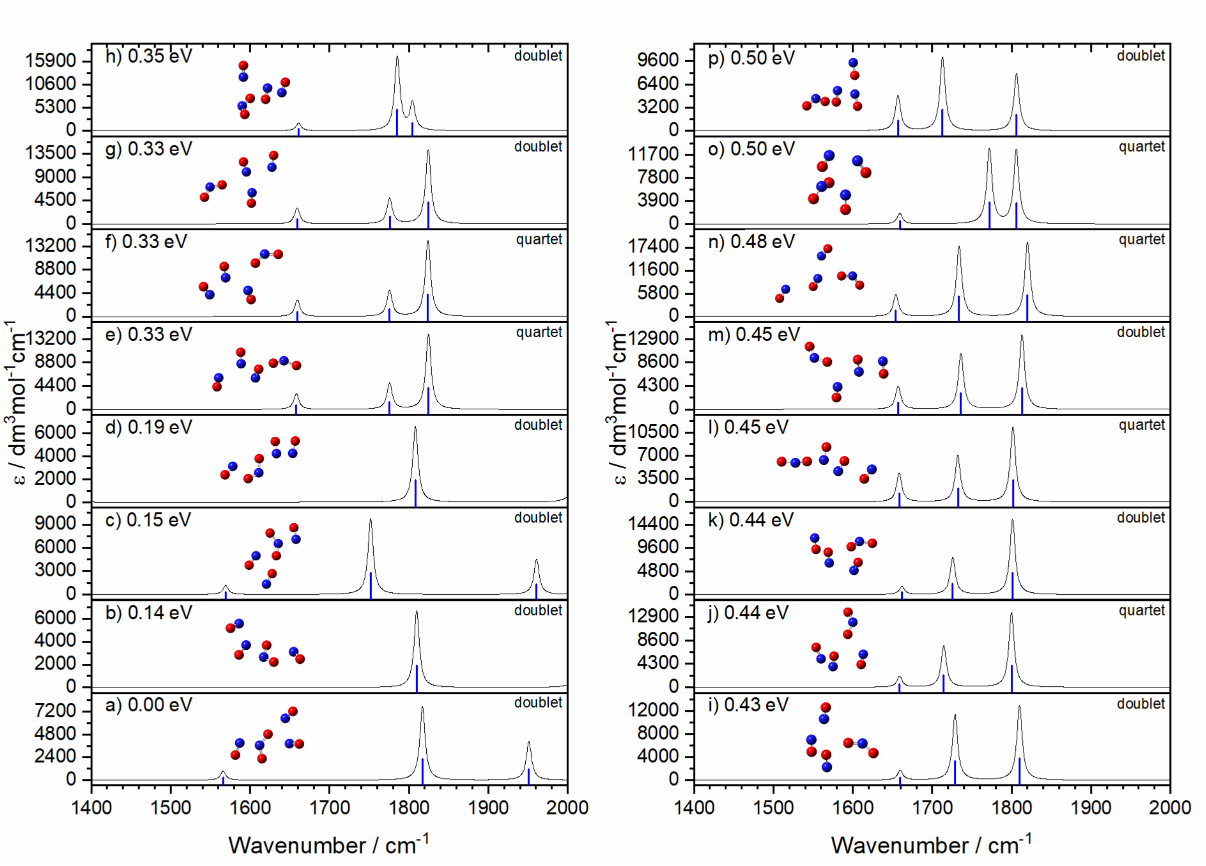

Figure S15: Simulated IR-PD spectra for the $[NO_2(NO)_3]^+$ complexes. Simulated spectra are generated from CAM-B3LYP/aug-cc-pVTZ calculations and scaled independently to the free NO stretching frequency (1876 cm^{-1}, SF = 0. 9268) and NO_2 asymmetric stretch (1616 cm^{-1}, SF = 0.9252).

References

(1) Mouhandes, A.; Stace, A. J. Infrared photofragmentation of “hot” and “cold” nitric oxide cluster ions. *J. Chem. Phys.* **1999**, *111* (21), 9517-9525,

(2) Odeneye, M. A.; Stace, A. J. Infrared photodissociation of $(NO)_n^+ \cdot X$ cluster ions ($n \leq 5$). *Phys. Chem. Chem. Phys.* **2005**, *7* (5), 998-1004,
